# Analysis of the Output of “Iranian Journal of Public Health” during 2015–2018

**Published:** 2019-02

**Authors:** Dariush D FARHUD

As usual to each New Year, the annual review of Iran J Public Health, hereby is presented for the year 2018. Besides, this editorial will compare the trend of whole publication process during 2015–2018.

**Fig. 1:**
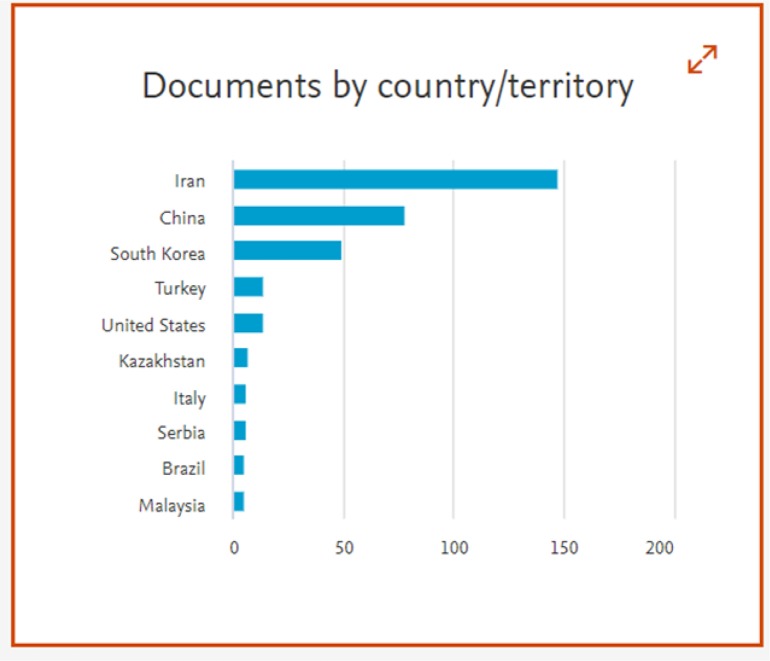
Distribution of papers published in IJPH based on country in 2018

**Fig. 2:**
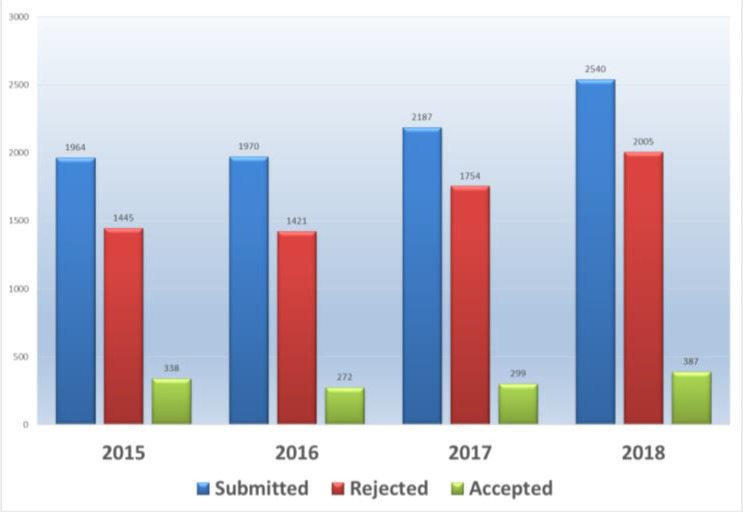
Total number of articles submitted during 2015–18 in the context of the frequency of submission, rejection and acceptance rate

The total number of manuscripts received during 2018 was 2540 from 60 countries. Of course, only the country of corresponding author was considered, so altogether much more countries we had in the panel. Again, Iran had the highest rate of submission, followed by China and South Korea (Table 1) (Fig. 1). Figure 2 presents total number of articles published during 2015–18 in the context of the frequency of submission, rejection and acceptance rate.

**Table 1:** Frequency of manuscripts received by Iran J Public Health during 2018 in terms of the frequency of submission, rejection and acceptance rate

**No.**	**Country**	**Submitted**	**Rejected**	**Accepted**
**1**	**Afghanistan**	1		
**2**	**Albania**	1	0	1
**3**	**Algeria**	1	-	-
**4**	**Armenia**	1	0	1
**5**	**Australia**	2	2	0
**6**	**Bangladesh**	4	1	2
**7**	**Belgium**	1	1	0
**8**	**Brazil**	13	9	3
**9**	**Bulgaria**	9	6	3
**10**	**Burkina Faso**	1	1	0
**11**	**Canada**	1	1	0
**12**	**Czech**	1	0	1
**13**	**China**	369	265	75
**14**	**Colombia**	1	0	1
**15**	**Cyprus**	5	-	-
**16**	**Egypt**	6	6	0
**17**	**Ethiopia**	1	-	-
**18**	**Ghana**	1	0	1
**19**	**Greece**	4	3	0
**20**	**Hong Kong**	1	-	-
**21**	**India**	21	21	0
**22**	**Indonesia**	61	53	8
**23**	**Iran**	1322	1071	172
**24**	**Iraq**	21	21	0
**25**	**Italy**	3	3	0
**26**	**Japan**	8	0	4
**27**	**Jordan**	8	8	0
**28**	**Kazakhstan**	16	9	6
**29**	**Korea**	96	49	37
**30**	**Kuwait**	2	2	0
**31**	**Lebanon**	1	1	0
**32**	**Macedonia**	1	0	1
**33**	**Malaysia**	43	27	13
**34**	**Mexico**	1	1	0
**35**	**Montenegro**	2	1	1
**36**	**Morocco**	11	8	3
**37**	**Myanmar**	1	1	0
**38**	**Nepal**	1	1	0
**39**	**Nigeria**	9	9	0
**40**	**Pakistan**	87	75	10
**41**	**Palestinian**	5	5	0
**42**	**Poland**	16	9	6
**43**	**Qatar**	1	1	0
**44**	**Romania**	26	22	4
**45**	**Russia**	6	4	0
**46**	**Saudi Arabia**	9	9	0
**47**	**Serbia**	29	19	7
**48**	**Slovakia**	8	0	8
**49**	**Slovenia**	1	0	1
**50**	**South Africa**	5	4	0
**51**	**Spain**	2	0	1
**52**	**Sri Lanka**	1	0	1
**53**	**Syria**	2	2	0
**54**	**Taiwan**	2	2	0
**55**	**Thailand**	4	3	1
**56**	**Tunisia**	9	8	1
**57**	**Turkey**	264	245	12
**58**	**Ukraine**	2	2	0
**59**	**United States**	7	5	2
**60**	**Vietnam**	2	2	0
	**Total**	2540	2005	387

Out of total submission of 2540 articles during 2018, 2005 articles were rejected after initial in-house evaluation or later peer review. Therefore the acceptance rate for this year was 15.2%.

In 2018, still we had unfortunately noticed some cases of plagiarism, which were treated according to the journal policy and COPE instructions. Normally, authors of minor cases of plagiarism are given a chance to amend their manuscripts precisely but major cases are rejected.

**Fig. 3:**
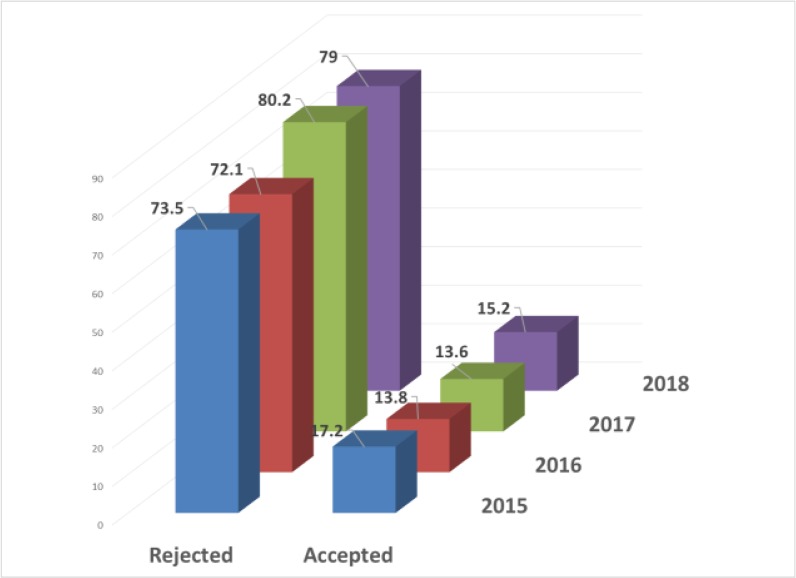
Total number of articles published during 2015–18 in terms of the percent of acceptance and rejection rate

The Journal continued its policy as for exact peer review rules including in-house evaluation followed by double blind peer review system. The reasons for rejecting a manuscript during in-house evaluation are various but the most important cases are out of scope cases, poor outcome, local studies, clinical contents etc. Figure 3, demonstrates the total number of articles published during 2015–2018 in terms of the percent of acceptance and rejection rate. It is worth mentioning that some manuscripts submitted during 2018, are still in the process of peer review so we have no idea of their destination. However, the rejection rate in 2018 was 79%.

**Fig. 4:**
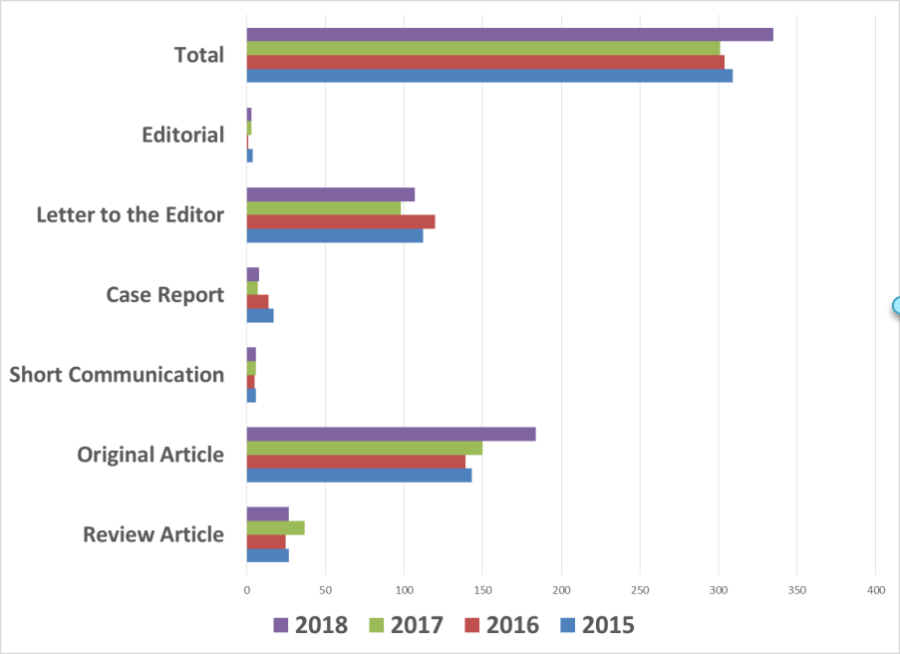
Total number of articles durng 2015–18 based on the type of published papers

The types of articles published during 2015–2018 are shown in Fig. 4. Accordingly, Original Articles had the highest rate of publication during the last four years.

Due to high flow of submitted manuscripts, in many cases, the authors were requested to change the format of “Original Article” to “Letter to the Editor”, which of course the merit of both formats remains the same.

According to http://www.scimagojr.com/, the H index of the journal is 24 until 2017. Besides, SCOPUS has reported the Site Score of the journal as 0.85 for 2017, besides SNIP as 0.6788.

**Fig. 5:**
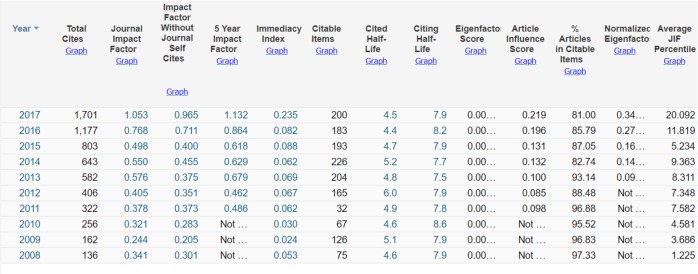
Key indicators announced by Clarivate Analytics (ISI) including Impact factor of IJPH during 2009–16

The good news for us and respected authors was that the Impact Factor announced by Clarivate Analytics (ISI) for the year 2017 was 1.053. In comparison to previous years, fortunately we had a great improvement on the journal quality. We should respect all referees and authors from all over the world to cooperate with us sincerely and caused their journal to further its aims. We do hope in near future, to improve this rate as much as possible (Fig. 5). This figure shows some key indicators reported by Clarivate Analytics during 2008–17.

